# Calcium Ion Channels in *Saccharomyces cerevisiae*

**DOI:** 10.3390/jof9050524

**Published:** 2023-04-28

**Authors:** Xiao-Yu Dong

**Affiliations:** College of Life and Health, Dalian University, Dalian 116622, China; dongxiaoyu@dlu.edu.cn

**Keywords:** calcium ion channels, *Saccharomyces cerevisiae*, gating mechanism, affecting factors, industrial application

## Abstract

Regulating calcium ion (Ca^2+^) channels to improve the cell cycle and metabolism is a promising technology, ensuring increased cell growth, differentiation, and/or productivity. In this regard, the composition and structure of Ca^2+^ channels play a vital role in controlling the gating states. In this review, *Saccharomyces cerevisiae*, as a model eukaryotic organism and an essential industrial microorganism, was used to discuss the effect of its type, composition, structure, and gating mechanism on the activity of Ca^2+^ channels. Furthermore, the advances in the application of Ca^2+^ channels in pharmacology, tissue engineering, and biochemical engineering are summarized, with a special focus on exploring the receptor site of Ca^2+^ channels for new drug design strategies and different therapeutic uses, targeting Ca^2+^ channels to produce functional replacement tissues, creating favorable conditions for tissue regeneration, and regulating Ca^2+^ channels to enhance biotransformation efficiency.

## 1. Introduction

As cell membrane proteins, calcium ion (Ca^2+^) channels carry out some key functions, including Ca^2+^ signal transduction, cell cycle, transport motility, and gene expression [1,2]. Different stimuli or stresses can induce the opening or closing of channel proteins distributed in the plasma membrane and various organelles. Additionally, any disruption in Ca^2+^ homeostasis can lead to alterations of certain essential functions in the cells.

As a model unicellular eukaryote, the budding yeast *Saccharomyces cerevisiae* is one of the most studied organisms worldwide, with its genome being thoroughly mapped both genetically and physically [3]. Moreover, *S. cerevisiae* provides a myriad of compatible plasmids and phages for genetic engineering. Notably, *S. cerevisiae* is the key microorganism in the industrial production of bioethanol, a clean, renewable, and sustainable alternative fuel [4]. As such, *S. cerevisiae* is widely used in theoretical research and production practice.

Ca^2+^ channels in *S. cerevisiae* act as the structural homologue and/or a functional analogue of the part of the Ca^2+^ channels in human or animal cells [3]. The deletion of genes encoding these channel proteins leads to an observable phenotype in yeast. Thus, *S. cerevisiae* is often used to introduce mutations in homologous genes associated with diseases or metabolic alterations in humans. Furthermore, various techniques for isolating and measuring channel activity, such as patch clamp, are well established in yeast. In this context, many studies have been conducted to understand the structure–function relationship of yeast proteins in disease or altered metabolism states. In this review, the development of novel regulation strategies for Ca^2+^ channels in *S. cerevisiae* is summarized comprehensively.

## 2. Types and Characteristics of Ca^2+^ Channels in *S. cerevisiae*

**Figure 1:**
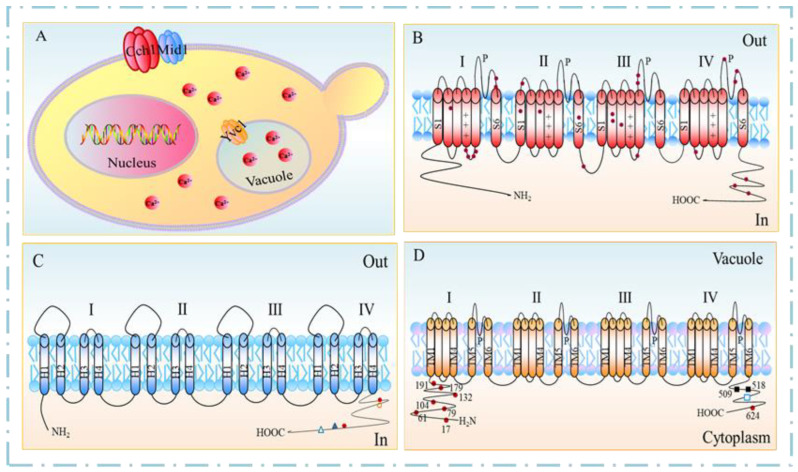
Calcium ion (Ca^2+^) channels in *Saccharomyces cerevisiae.* (**A**) Channel localization. (**B**–**D**) Predicted topologies of Ca^2+^ channels Cch1, Mid1, and Yvc1; the plus sign indicates the positively charged amino acids; solid circle indicates the cysteine (Cys) residues (Cch1, Yvc1) or Cys-rich regions (Mid1); hollow circle indicates the EF-hand-like motif of helix–loop–helix structure; solid triangle indicates the CKII phosphorylation motif; hollow triangle indicates the sheet–turn–sheet structure; solid square indicates the hydrophobic patch between the amino acid residues 509 and 518; hollow square indicates the negatively charged cluster _573_DDDD_576_.

Based on their activation mechanisms, Ca^2+^ channels in *S. cerevisiae* are classified into three types: (i) Cch1 of the voltage-gated calcium channel (VGCC); (ii) Mid1 of the stretch-activated calcium channel (SACC); and (iii) Yvc1 of the transient receptor potential (TRP) channel [5,6,7] (Figure 1A). Cch1 and Mid1 are located in the cell membrane, whereas Yvc1 is located in the vacuole membrane. Ca^2+^-permeable channels regulate the passive flow of Ca^2+^ across the plasma membrane into the cytoplasm.

### 2.1. VGCC

In *S. cerevisiae*, the gene *CCH1* encodes the Cch1 protein, comprising 2039 amino acid residues with a molecular weight of 234.6 kDa, which has been predicted as a multiple membrane-spanning transmembrane protein (Figure 1B). Cch1 contains four structurally similar hydrophobic domains (I-IV), with each domain having six transmembrane domain (TMD) segments [8]. All four domains contain the amino acid residue indicative of the Ca^2+^-selective P segment (channel specificity), whereas three of the four domains (i.e., II, III, and IV) contain a highly conserved glutamate residue, which plays a critical role in Ca^2+^ coordination. Each S4 segment of domains I, II, and III contains repeated motifs of a positively charged amino acid residue, followed by two hydrophobic amino acid residues (voltage dependence).

Cch1 is a homolog of the pore-forming subunit α1 of the dihydropyridine-sensitive (L-type) family of mammalian VGCCs [9], retaining several key features, including the size, topology, and domain structure of α1 subunits. Cch1 contains a total of 28 cysteine residues, of which four are found in an internal loop connecting TMD4 and TMD5 of segment I, and two at each pore region of segment III and segment IV are highly conserved across species, from yeast to humans [10].

The overall amino acid sequence similarity between Cch1 and α1 subunits of L-type VGCC in mammal cells is low, i.e., 24% for Cch1 compared to a mammalian L-type VGCC. Generally, the mammalian VGCCs contain 19 lysine and arginine residues located in the voltage sensing S4 domains, whereas Cch1 and its homologs in fungi contain 11 to 12 lysine and arginine residues at these corresponding sites [9,11], which can potentially alter the membrane voltage by changing the response speed and sensitivity. Additionally, in mammalian voltage-gated calcium (Ca_v_) channels, a Ca^2+^-binding site contributing to the ionic selectivity is formed by a quartet of pore-localized glutamate residues (EEEE locus), one from the selectivity filter region of each of the four domains [12]. Moreover, each of the Cch1 channel homologues identified in the fungi possesses a similarly placed acidic motif but with three acidic residues rather than four [13].

### 2.2. SACC

The gene *MID1* encodes Mid1, a transmembrane polypeptide comprising 548 amino acid residues with a molecular weight of 61.5 kDa. As the major and independent Ca^2+^ entry route, Mid1 is mostly involved in Ca^2+^ uptake at a low Ca^2+^ concentration of 100 μM and mating. The Mid1 channel has four hydrophobic regions, corresponding to the amino acid residues of 2–22, 92–111, 337–356, and 366–388, namely, H1, H2, H3, and H4, respectively (Figure 1C).

Mid1 shares no extensive similarity with any known proteins. The H1 segment is a signal sequence, whereas the H2 and H4 segments are partially similar to the transmembrane segments of known ion channels, and the H3 segment is similar to that of the pore-forming regions of several cation channels [6]. In the H2 segment, the glycine residue plays a vital role in channel gating [14]. According to the protease protection experiments on intact cells, the region between the H3 and H4 segments is often located outside the plasma membrane, suggesting that the Phe356 residue would be located outside the plasma membrane. The hydrophobicity of the Phe356 residue is essential for viability maintenance and Ca^2+^ uptake, while its size significantly affects viability maintenance [15].

Mid1 is a plasma and endoplasmic reticulum (ER) membrane protein with 16 N-glycosylation sites and a C-terminal cysteine (Cys)-rich region comprising 12 Cys residues [16]. The Cys-rich region is located in the domain between the H4 segment and the Thr526 residue, with at least three motifs, including a putative casein kinase 2 phosphorylation motif, a sheet–turn–sheet structure, and an EF-hand motif of helix–loop–helix structure [14]. The sheet–turn–sheet structure significantly contributes to the complementing ability and Ca^2+^ uptake activity, whereas the EF-hand motif contributes to the sites of interaction with other proteins or Mid1 itself.

In ER, Mid1 is located as a 200-kDa oligomer by covalent cysteine bonding, probably through the Cys-rich region. There is evidence that Mid1 is N-glycosylated in the ER, and then transported to the plasma membrane. The hypothesis is supported by the presence of a putative N-terminal signal peptide and several potential transmembrane (TM) α-helices [17].

### 2.3. TRPY1

The gene *YVC1* encodes *S. cerevisiae* vacuolar transient receptor potential yeast1 (TRPY1, also known as yeast vacuolar conductance 1, or Yvc1). Yvc1 comprises 675 amino acid residues with a molecular weight of 78 kDa. As an ion channel responsible for the efflux of vacuolar Ca^2+^ to the cytoplasm, Yvc1 is the only member of the TRP superfamily expressed in *S. cerevisiae* [18]. As a low-copy-number protein, the number of Yvc1 molecules per cell during the exponential growth phase is approximately 1300 [19]. Additionally, the whole vacuole currents, combined with a single-channel recording, show at least 100 channels per vacuole [5].

Similar to all TRP members, Yvc1 contains six predicted TMDs with cytosolic N- and C-termini and a hydrophobic pore region located between TMD5 and TMD6. TMD6 forms the part of the ion conduction pathway and takes part in the deactivation of cation channel gating [5] (Figure 1D). The short amino acid sequence motif, VILLNILIALY, between the residues 448–458 in TMD6 in *S. cerevisiae*, is highly conserved in the corresponding TMD6 sequence of most mammalian TRPs. Additionally, the C-terminus of Yvc1 contains a cluster of four acidic residues involved in Ca^2+^-dependent regulation, which is analogous to the Ca^2+^-binding bowl in the big-conductance Ca^2+^-activated K^+^ (BK) channels [20]. Yvc1 contains nine cysteine residues, eight of which are cytoplasmic, and only Cys-343 is located within TMD2. Among these cysteine residues, only Cys-191 is conserved across yeast and fungi, and three cysteine residues of Cys-17, Cys-79, and Cys-191 are specifically glutathionylated. It is reported that glutathione depletion in yeast leads to the activation of Yvc1 [21]. Recently, the full-length cryoelectron microscopy structure of Yvc1 has been elucidated, which is consistent with most previously studied TRP channel proteins but has distinct structural folds in the cytosolic N and C termini [22].

## 3. Gating Mechanisms of Ca^2+^ Channels

The open–close status of Ca^2+^ channels is key to achieving the structure–function relationship. Some physiological activities of cells, such as the generation of pacemaker potential, the contraction of the muscle cells, the plateau phase of the cardiac action potential, and gene expression, are mainly mediated by fine-tuning Ca^2+^ entry through Ca^2+^ channels [23]. Moreover, the opening (activation) and closing (inactivation) of various types of channels depend on different gating mechanisms.

### 3.1. Voltage-Dependent Gating

**Figure 2:**
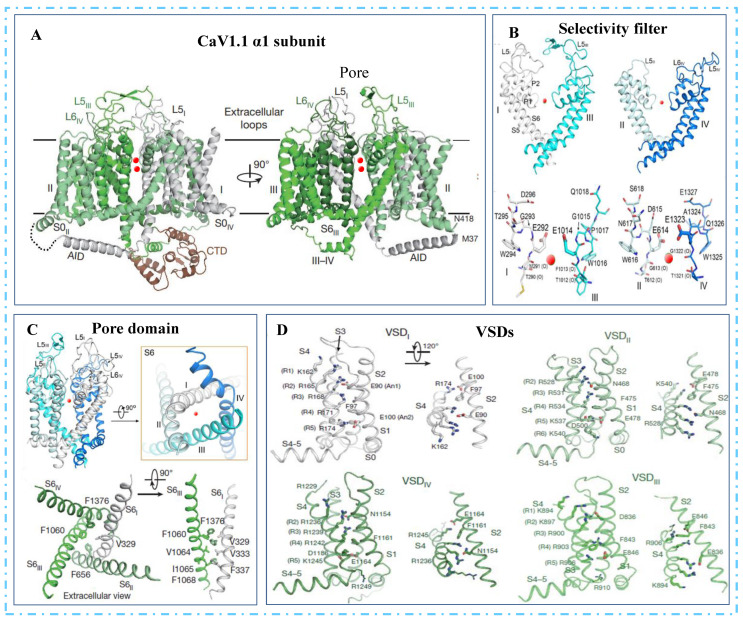
Structure of the α_1_ subunit in Ca_v_1.1 [25,26]. (**A**) Overall structure of α_1_ subunit; CTD, C- terminal domain; red spheres indicate the tentatively assigned calcium ions (Ca^2+^) in the selectivity filter vestibule. (**B**) Structural elements of the selectivity filter. (**C**) Four-fold pseudo-symmetry of the pore domain; loops between S5 and P1 helices and between P2 and S6 helices are shown as L5 and L6 loops, respectively. (**D**) Structures of the four voltage-sensing domains (VSDs). Gating charges on helix S4 and the occluding phenylalanine residue on S2 are shown as sticks.

It is reported that the gating mechanism of Cch1 is consistent with the α-subunit of L-type VGCC in mammalian cells in terms of sequence similarity [24]. The atomic model of the α1-subunit comprises the pore domain, the four voltage-sensing domains (VSDs), the intracellular α1-interacting domains (AID)-containing helix between the I and II domains (the I-II helix), and the intact III-IV linker (Figure 2A). The selectivity filter (SF) in the pore domain located at the extracellular side of TMD contains the EEEE locus (Glu292/614/1014/1323) [25,26] (Figure 2B). The selectivity filter extends to the channel cavity surrounded by a tetrameric arrangement of segments S5 and S6 (Figure 2C). In all four VSDs, the gating charges are aligned on one side of the 310 helices of S_4_ segments. Moreover, the R5 residues and R6_II_ are underneath, whereas the R1-R4 residues are above the conserved occluding Phe residue in the charge transfer center, representing the depolarized or “up” conformation of VSDs (Figure 2D). In contrast, the negative residue on S3 responsible for the charge transfer center is located in the VSD_II_ and VSD_IV_, but not in the other two VSDs. Collectively, these may indicate a potentially inactivated state of Ca_v_1.1.

VSDs and the pore domain are mainly lined by the S4–S5 linkers, which directly interact with all four S6 segments through the residues G432 (IS6), A780 (IIS6), G1193 (IIIS6), and A1503 (IVS6), designated as the G/A/G/A ring. Thus, mutations in the G/A/G/A residues affect the VSDs movement [27], in particular, the S6 helices form the activation gate at the intracellular end of the TMD. In the closed state, the pore-linking S6 helices converge at the intracellular flank to obstruct ion permeation. Upon opening, the intracellular ends of the S6 helices diverge from one another, thereby creating a wide opening to enable ion passage [28]. 

It is reported that all eight conserved extracellular Cys residues are associated with the Cch1 activity. However, replacing the Asn^1066^ residue with a negatively charged amino acid might change the conformation of the cytoplasmic II-III interdomain loop, thereby turning Cch1 into a closed structure prone to opening even in the absence of stimuli [8]. Conversely, replacing Gly^1265^ in the S2–S3 linker of domain III perturbs the spatial arrangement between S2 and S3, resulting in a loss of activity of the Cch1 [29]. Additionally, the residue Pro^1228^ is located in the extracellular S1-S2 linker of domain III and is well conserved across fungi and humans. Therefore, the mutation in this residue leads to a significant loss of activity of Cch1 [8].

### 3.2. Stretch-Activated Gating

Mid1 is the only known SACC in *S. cerevisiae*. So far, many mutation experiments have been employed to investigate the structure–function relationship of Mid1. Studies have shown that the mutant without the whole H3 or H4 segment and H3De or H4De loses the complement capacity in the lethality and shows low Ca^2+^ accumulation activity. In particular, the C373D and C373R proteins completely lost the Mid1 function related to viability and Ca^2+^ accumulation [6]. Therefore, segments H3 and H4 are essential for the Mid1 function. A recent study has proved that the Cys-498 residue at the C terminus of Mid1 is essential for Ca^2+^ channel activity [30].

### 3.3. Transient Receptor Gating

Yvc1, as a prominent voltage-dependent channel, possesses two gating systems for incorporated channels [31]. Gate one responds relatively fast to voltage fluctuations, and the probability of opening decreases as the potential tends towards positive but increases in the presence of the stilbene derivative 4,4-diisothiocyanato-stilbene-2,2-disulfonic acid (DIDS) at the cytoplasmic flank. In contrast, gate two responds relatively slowly to voltage fluctuations, and the probability of opening increases as the voltage potential tends toward positive. Moreover, Ca^2+^ is required at the cytoplasmic flank, which shifts voltage dependence towards negative.

Furthermore, Yvc1 is a mechanosensitive channel which can be activated to release Ca^2+^ from the vacuole to the cytoplasm by a transient osmotic force. The increase in osmolarity near the vacuole causes the shrinkage of the vacuole within seconds, inducing water withdrawal from the cytoplasm and vacuole, and this temporary osmotic imbalance produces an osmotic pressure across the vacuolar membrane [32]. It is reported that the membrane is stretched along its plane upon exerting pressure, either mechanical or osmotic, on the membrane regardless of the direction [33], which forces Yvc1 molecules into an open conformation. Eventually, the net water flux and osmotic pressure across the membrane recedes and ceases [32].

In a previous study, a novel structure–function model of mechanical force and cytoplasmic Ca^2+^ activating Yvc1 in parallel has been reported in *S. cerevisiae* [20]. In this model, the mechanical force acts through the membrane-associated domains, and Ca^2+^ binds to the negatively charged residues in the cytoplasmic domains, generating energy that reaches the gate in parallel. Recent studies have elucidated that Yvc1 gating in yeast is regulated by Ca^2+^, PI(3)P lipids, and membrane stretch. Furthermore, the mechanical force induces the release of Pl(3)P lipids from their binding sites and alters channel conformation, thereby promoting pore opening and Ca^2+^ flux from the vacuole to the cytosol [22].

### 3.4. Factors Affecting Ca^2+^ Channel Gating States

Several environmental factors can affect Ca^2+^ channel gating, including Ca^2+^ channel antagonists, aromatic compounds, oxidative stress, osmotic pressure, alkaline stress [34], temperature stress [35], cold stress [36], etc. Due to space limitations, only four main factors affecting Ca^2+^ channel gating have been discussed in this review (Table 1).

## 4. Applications of Ca^2+^ Channels in *S. cerevisiae*

**Figure 3:**
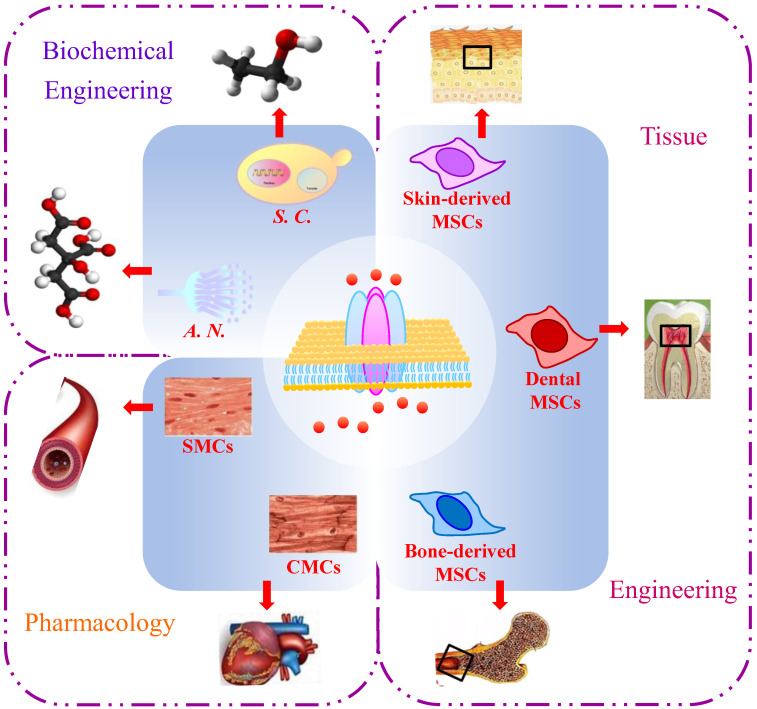
Applications of calcium ion (Ca^2+^) channels in *Saccharomyces cerevisiae*. SMCs, smooth muscle cells; CMCs, cardiac muscle cells; *S. C.*, *Saccharomyces cerevisiae*; *A. N.*, *Aspergillus niger*; MSCs, mesenchymal stem cells.

Studying the gating mechanisms and regulators of Ca^2+^ channels will significantly contribute to various applications in pharmacology, tissue engineering, and biochemical engineering (Figure 3).

### 4.1. Pharmacology

Ca^2+^ channels are essential targets for pharmaceutical research due to their implication in various diseases. *S. cerevisiae* has been widely used to study the pharmacological mechanism of drugs, including alkoxy-phenols of anti-tumor drugs and dihydropyridines of calcium channel blockers, by exploring biochemical alterations [7] due to the similarity between Cch1p and L-type VGCCs. Drugs in the dihydropyridine calcium channel blocker class, e.g., nifedipine and nimodipine, are likely to induce a permanent opening of Ca^2+^ channels by binding to the subunit α_1_, thus generating a non-controlled influx of Ca^2+^ into cells, which leads to a collapse of the membrane potential in *S. cerevisiae* [39]. Amiodarone, one of the most effective antiarrhythmic agents for chronic arrhythmias, can stimulate the activity of Mid1 but not Cch1. It is reported that a sustained increase in Ca^2+^ influx can cause cardiac arrhythmia. Thus, exploring the mechanism of Ca^2+^ homeostasis in yeast upon amiodarone inducement can provide insights into cardiac diseases [50].

*S. cerevisiae* has also been used as a model in studies exploring channelopathies. Particularly, Yvc1 is used as a model to study mucolipidosis type IV [3]. Moreover, the response of Cch1 to stress within the secretory pathway is consistent with the phenomenon observed in the mammalian cells, which is coupled to Ca^2+^ influx. Therefore, it is speculated that fungal and animal cells use a similar mechanism to trigger Ca^2+^ influx in response to secretory stress. Moreover, disrupting the function of presenilin-1 in the endoplasmic reticulum of neurons might affect several aspects of Ca^2+^ influx and signaling [51]. Accordingly, the study of Ca^2+^ channels in the yeast provided insights into familial Alzheimer’s disease [52].

### 4.2. Tissue Engineering

Current research on tissue engineering is mainly focused on producing functional replacement tissues and developing favorable conditions for tissue regeneration. L-VGCCs are significant for the osteogenic, myogenic, and neural differentiation of several types of stem cells. Thus, these L-VGCCs are involved in the function regulation of various stem cell types, including mesenchymal stem cells (MSCs) from the bone marrow, the oral cavity, adipose, and skin tissues, etc. Moreover, the corresponding regulatory mechanisms have been shown to influence cell proliferation and multipotent differentiation [53].

Recent studies have successfully demonstrated that targeting L-VGCCs modulates neurogenic activities due to the indispensable role of L-VGCCs in neurogenesis. Additionally, exposure to extremely low-frequency electromagnetic fields (i.e., 50 Hz) could increase the expression of VGCCs and modulate their function, especially Ca_V_1.2 and Ca_V_1.3 [54]. Ultimately, large-amplitude Ca^2+^ influx and a higher percentage of responsive neurons result in increased cell proliferation and neural differentiation. Overall, these results provide a reference for further research on tissue regeneration and development and broadening the potential applications of L-VGCCs in tissue engineering in the future.

### 4.3. Biochemical Engineering

It is well known that Ca^2+^ can significantly improve cell metabolism, thereby increasing the yield of the target product in microbial fermentation. Therefore, regulating the Ca^2+^ channels is a potential strategy for enhancing microbial productivity in green bio-manufacturing [45]. To date, very few reports have explored the regulation of Ca^2+^ channels in industrial fermentation.

It has been reported that ethanol production in *S. cerevisiae* was improved under the treatment of air cold plasma. In the further study, it has been explored that the Cch1/Mid1 in the cell membrane and Yvc1 in the vacuole membrane could be activated opening by the air cold plasma, and the increased cytoplasmic Ca^2+^ promotes the expression and activity of Pma1 H^+^-ATPase. Consequently, the cofactor metabolism from ATP and NADH is disturbed, enhancing ethanol production by *S. cerevisiae* [45]. In immobilized fermentation, biofilm plays a key role in improving industrial fermentation efficiency. In fact, Ca^2+^ channels CchA and MidA have been shown to upregulate biofilm formation in *Aspergillus niger* [55]. Therefore, regulating the Ca^2+^ channels in industrial microbes could provide a novel and promising strategy for bioprocess enhancement.

While elucidating the gating mechanism, exploring the crystal structures of Cch1 and Mid1 in *S. cerevisiae* is still challenging. Moreover, cloning of the *CCH1* gene has been considered a major challenge due to its toxicity to *E. coli*. Consequently, direct studies on the *CCH1* channel activity in the heterologous expression systems are inexecutable. In this regard, Vu et al. established a novel approach in which the shuttle plasmid *CCH1*-GFP, prepared in vitro and propagated in yeast, was successfully expressed in the HEK293 mammalian cell line [56]. This method has significantly increased the possibility of cloning and expressing Ca^2+^ channel genes in *S. cerevisiae*.

In the author’s research group, studies on Ca^2+^ channels in *S. cerevisiae* are currently ongoing. Moreover, the properties of genes and proteins related to the three Ca^2+^ channels have been analyzed by bioinformatics tools, with certain functional fragments of these genes being cloned, expressed, and purified successfully [57]. The used methods and obtained data in these studies will lay a theoretical and technical foundation for the further exploration of the crystal structures of Cch1 and Mid1 in *S. cerevisiae*.

## 5. Conclusions

The regulation of Ca^2+^ channels has emerged as a promising technology in the field of novel drug design, disease treatment, tissue regeneration, and microbial fermentation. The increasing amount of ongoing research on the regulation mechanisms of Ca^2+^ channels will significantly contribute to the rapid development of this promising technology. The present review provides a comprehensive summary of the protein composition, channel conformation, gating mechanism, and practical applications of three types of Ca^2+^ channels in *S. cerevisiae*, namely, Cch1, Mid1, and Yvc1, which have been considered as important models forscientific studies. Overall, this review will provide a reference for further studies and broaden the possible applications of Ca^2+^ channels in various fields.

## Figures and Tables

**Table 1:** Environmental factors affecting calcium ion (Ca^2+^) channel gating.

Affecting Factors	Reagents	Calcium Ion Channels	Effects	Gating Mechanism	Applications	References
Calcium ion (Ca^2+^) channel antagonists/blockers	Amiodarone, amlodipine, diltiazem, nicardipine, nifedipine, nimodipine, nitrendipine, verapamil	Cch1, Cch1-Mid1, or Mid1	Blocking calcium channels or stimulating channel opening	Binding to the special sites on Ca^2+^ channels for blockage	Clinical treatment of hypertension, coronary heart disease, and cardiac arrhythmia	[7,37,38,39]
Aromatic compounds	Carvacrol, eugenol, indole, methylated, propylparaben, quinolone, thymol	Cch1 or Yvc1	Improving cell tolerance or mediating the Ca^2+^ increase in the cytoplasm	Competing with the aromatic residues of the channel proteins for lipid anchors or generating force profile in the bilayer for open conformation	Antifungal drugs	[40,41,42,43]
Oxidative stress	Chloramine T, dithiothreitol, hydrogen peroxide, 2-mercaptoethanol, mitochondrion, N-ethylmaleimide, plasma discharge, reduced glutathione, *β*-phenylethylamine, *tert*-butylhydroperoxide (*t*BOOH)	Cch1, Mid1, or Yvc1	Affecting activity, expression, open-time probability, or trafficking	Specific glutathionylation of cysteine residues in the pore forming region; oxidation of special amino acids; change in the channel conformation	Experiments on channel functions or metabolic regulation	[10,44,45,46,47]
Osmotic pressure	Ethanol, hexose (glucose and galactose), LiCl, NaCl, sorbitol	Cch1, Mid1, or Yvc1	Channel opening or improving ethanol tolerance	Membrane perturbation, vacuolar shrinkage and deformation, or activation of the PKC1 pathway	Industrial microbial fermentation	[32,48,49]

## Data Availability

Data sharing is not applicable to this article as no datasets were generated or analyzed in the course of the current study.
